# Prices, Availability and Affordability of Medicines with Value-Added Tax Exemption: A Cross-Sectional Survey in the Philippines

**DOI:** 10.3390/ijerph17145242

**Published:** 2020-07-21

**Authors:** Krizzia Lambojon, Jie Chang, Amna Saeed, Khezar Hayat, Pengchao Li, Minghuan Jiang, Naveel Atif, Gebrehaweria Kassa Desalegn, Faiz Ullah Khan, Yu Fang

**Affiliations:** 1 Department of Pharmacy Administration and Clinical Pharmacy, School of Pharmacy, Xi’an Jiaotong University, Xi’an 710061, China; krizlambojon@gmail.com (K.L.); jiechang@xjtu.edu.cn (J.C.); dr.amnasaeed92@gmail.com (A.S.); khezar.hayat@uvas.edu.pk (K.H.); lipengchao1996@stu.xjtu.edu.cn (P.L.); jiangmh2017@xjtu.edu.cn (M.J.); naveel_atif87@hotmail.com (N.A.); desalegnkassa188@gmail.com (G.K.D.); fkhan@bs.qau.edu.pk (F.U.K.); 2 Center for Drug Safety and Policy Research, Xi’an Jiaotong University, Xi’an 710061, China; 3 Shaanxi Centre for Health Reform and Development Research, Xi’an 710061, China; 4 Research Institute for Drug Safety and Monitoring, Institute of Pharmaceutical Science and Technology, China’s Western Technology Innovation Harbor, Xi’an 710061, China; 5 Institute of Pharmaceutical Sciences, University of Veterinary and Animal Sciences, Lahore 54000, Pakistan

**Keywords:** drugs, generic, private sector, costs and cost analysis, health services accessibility, World Health Organization, taxes, Philippines

## Abstract

Background: Developing countries, such as the Philippines, started implementing policies to improve access to medicines, which is a vital step toward universal healthcare coverage. This study aimed to evaluate the prices, availability and affordability of prescribed medicines for diabetes, hypercholesterolemia and hypertension with the exemption of 12% value-added tax in the Philippines. Methods: The prices and availability of 50 medicines were collected in August 2019 from 36 public and 42 private medicine outlets in six regions of the Philippines, following a modified methodology developed by the World Health Organization and Health Action International. Availability is reported as the percentage of outlets in which the surveyed medicine was found at the time of visit. Medicine prices are expressed as median unit prices (MUPs) in Philippine Peso. Affordability is calculated based on the number of days’ wages required for the lowest-paid unskilled government worker to purchase a monthly treatment. Results: The mean availability of surveyed medicines was low in both public and private sectors, with 1.3% for originator brands (OBs) and 25.0% for lowest-priced generics (LPGs) in public outlets, and 34.7% and 35.4% in private outlets, respectively. The MUP of medicines were higher in private outlets, and OBs have higher unit price compared to the generic equivalents. Treatments with OBs were unaffordable, except for gliclazide, but the affordability of most LPGs is generally good. Conclusion: Access to medicines in both sectors was affected by low availability. High prices of OBs influenced the affordability of medicines even with tax exemption. A review of policies and regulations should be initiated for a better access to medicines in the Philippines.

## 1. Introduction

Non-communicable diseases (NCDs), primarily cardiovascular diseases, diabetes, cancer and chronic respiratory diseases, are the foremost causes of death worldwide [1]. The impact of NCD burden is recognized as a major challenge for sustainable development. In the 2030 Agenda for Sustainable Development Goal (SDG) 3, target 3.4 focuses on the reduction in premature NCD mortality by a third [2,3]. The importance of access to effective, quality and affordable essential NCD medicines as a vital intervention in preventing morbidity and mortality is reflected in SDG 3.8, as well as by the Universal Health Coverage [4].

In the Philippines, 67% of all deaths were estimated to be attributed to NCDs [5]. Cardiovascular diseases remain the leading cause of mortality in the country, and diabetes ranks at sixth [6]. Access to essential medicines for these primary NCDs is insufficient in the country. The previous World Health Organization/Health Action International (WHO/HAI) methodology survey conducted in 2009 revealed the low availability and high prices of essential medicines in the Philippines. The prices of originator brands were more than 30 times and the prices of generic medicines were about ten times the international reference price in both public and private sectors [7].

The Philippine government took several measures to improve the access to medicines, such as promotion of generic drugs, the Cheaper Medicines Act with price-capped medicines under the maximum drug retail prices (MDRP) and the government-mediated access prices (GMAP) programs, and the Department of Health (DOH) medicines access programs [8,9]. In spite of these actions, a survey conducted by the Center for Legislative Development in 2010, found that poor people still find it challenging to purchase the standard treatment of medicines [10]. In addition, the household out-of-pocket (OOP) health expenditure increased by 21% from 2009 to 2012, with nearly 50% of the OOP being attributable to the cost of medicines [11]. Similar expenditures were noted in both 2016 and 2017. The household OOP payment took 54% of the total health expenditure in both years, 27.5% of OOP went to pharmacies in 2016 and it increased by 50.1% in 2017 [12,13]. The Philippine Institute for Development Studies has reported in 2018 the high cost of medicines in the country [14].

High prices of medicines is the primary barrier to access [15]. Affordable medicine prices can be achieved by applying measures such as reducing or removing taxes, margins and tariffs [1]. Value-added tax (VAT) on medicines varies in different countries from zero to 19% globally, while it is 12% in the Philippines [15,16]. On 1 January 2019, the Philippine government formally implemented the Section 109-AA of Republic Act 10693, or known as “Tax Reform for Acceleration and Inclusion Law” [17]. Pursuant to this section, the sales of prescribed medicines for diabetes, hypercholesterolemia and hypertension, listed in the formulary approved by the Food and Drug Administration (FDA) of the Philippines, have been exempt from the 12% value-added tax [18]. The coverage and scope of this section apply to the sales of the above-described medicines by domestic manufacturers, distributors, wholesalers and retailers in its final dosage form. Nonetheless, imported medicines are still subjected to the 12% VAT [18].

Although many developing countries have removed or reduced the taxes on medicines, the actual effect of these policies on improving access to medicines and reducing final prices paid by patients has been largely unclear. For example, Peru removed the VAT for anticancer and antiretroviral drugs in 2001 [15], but only a small change in retail prices was found after the policy, as it may be influenced by increased mark-up in the supply chain [16]. Waiving of taxes does not guarantee lower prices to patients unless supporting regulation and monitoring is applied simultaneously. In this study, we aimed to evaluate the access to 50 prescribed medicines for diabetes, hypercholesterolemia and hypertension in the Philippines, applying the policy of 12% VAT exemption on its early stage of implementation, using a modified WHO/HAI methodology for surveying medicine prices, availability and affordability. Previous work was conducted in 2009 that mainly focused on essential medicines [7] and to the best of our knowledge, no updated studies in the Philippines were then published using the same methodology.

## 2. Methods

This study was a cross-sectional survey of availability, prices and affordability of 50 prescribed medicines for diabetes, hypercholesterolemia and hypertension. Data were collected from medicine outlets in both public and private health sectors in the Philippines from 1 August 2019 to 30 August 2019, following a modified methodology developed by the WHO and HAI [19].

### 2.1. Study Setting and Sampling

The Philippines is an archipelago categorized into three island groups, namely: Luzon, Visayas and Mindanao. Each island group is divided into regions with 8, 3 and 6 regions, respectively. The selection of regions as survey areas were based on the geographical distribution and socioeconomic status. A total of six survey areas (three regions in Luzon, one region in Visayas, and two regions in Mindanao) were included as representative of the whole country (see Table A1 in Appendix A).

Systematic sampling was used to select medicine outlets, wherein each survey area is comprised of five public and five private medicine outlets. One main public hospital with outpatient pharmacy was identified, and an additional four public medicine outlets were randomly selected within three hours’ drive from the main hospital. The private sector was identified by selecting private hospitals or retail pharmacies closest to the surveyed public medicine outlets. Back up outlets in each sector were visited when fewer than 50% of surveyed medicines were found. This resulted in a total of 78 health facilities, 36 public medicine outlets and 42 private hospital/retail pharmacies being included.

### 2.2. Medicines Selection

A total of 50 medicines were included in the study. The selection was based on the Philippine National Formulary 8th Edition Essential Medicines List (PEML) [20], the WHO/HAI Global Core List of Essential Medicines [19], the WHO Model List of Essential Medicines 2017 [21] and the “List of VAT-exempt Diabetes, High-Cholesterol and Hypertension Drugs” [22] published by the FDA Philippines. Among the surveyed medicines, four were global core essential medicines, 21 were part of the model list of the WHO, and 37 were listed on the PEML. Thirty antihypertensive drugs, five lipid-lowering agents and 15 antidiabetic drugs were included in the study. For each medicine, data on the originator brand and lowest-priced generic were collected.

### 2.3. Data Collection and Entry

Data collection was conducted through on-site visits in each public and private health facility. Data were obtained by a team of two well-trained collectors (licensed pharmacists) in each survey area. Collectors recorded the availability and prices of 50 medicines from the responsible personnel in every health facility, using a data collection form. The development of the survey form was based on the WHO/HAI template. Data were collected on both the originator brand and the lowest-priced generic of every surveyed medicine.

Medicines were marked as “available” after they were physically seen and checked by the data collectors. Price data were recorded for medicines in stock on the day of the survey. All data were entered in the WHO/HAI Excel Workbook by double entry technique, and the workbook’s auto checker was used to facilitate the verification process.

### 2.4. Data Analysis

Medicine availability is calculated as the percentage (%) of outlets per sector that had stock of the surveyed medicine at the time of visit, regardless of the amount available. Across the medicines, the mean availability was assessed for the originator brand and lowest-priced generic equivalent. Percentages were presented to describe the availability of medicines using the following ranges: not available (0%), very low (<30%), low (30–49%), fairly high (50–80%), and high (>80%) [23]. The WHO has set a cutoff point of 80% as the target availability of medicines [24].

In the WHO/HAI methodology, the structure of the health system should be considered when developing an analysis of the medicine availability in the public sector. The structure of the health system consists of three levels of care: primary, secondary and tertiary. Primary care is the first point of contact with the health system, secondary care refers to specialized ambulatory health services and tertiary care refers to regional hospitals with services of high complexity [19]. Surveyed medicines were recommended to be categorized based on the expected availability at a certain level of care in the public health sector. However, in this study, we were not able to categorize the medicines. Therefore, availability analysis across the different levels of care in the public health sector was not done.

Median unit price (MUP) of surveyed medicines was presented in local currency (PHP; Philippine peso). If medicines found in fewer than three outlets, MUP was not calculated. Unit price is defined as the price of an individual tablet, capsule, milliliter, gram or dose, except for insulin where the unit price is in 10mL vial [25]. The normality and homogeneity of variance were tested using SPSS 22.0. Either, parametric (independent *t* test) or non-parametric (Mann–Whitney U test) analysis was performed to compare the medicine prices between the public and private sectors [26]. A *p*-value of <0.05 was considered statistically significant.

Affordability is measured as the number of days’ wages required for the lowest-paid unskilled government worker to purchase one course of treatment or for a monthly treatment in case of chronic conditions. Twenty medicines were included in the analysis: four global core list medicines, ten medicines included in the WHO Model list, and six supplementary drugs from PEML. The surveyed medicines were considered as maintenance drugs for patients diagnosed with diabetes, hypercholesterolemia and hypertension. Thus, the monthly treatment of the surveyed medicines was utilized. The 2019 salary of the lowest-paid government unskilled worker in the Philippines is 369 PHP per day [27], which is equivalent to 7.22 USD (1 USD = 51.1218 PHP, August 2019) [28]. 

### 2.5. Ethical Approval and Consent to Participate

This study was reviewed by the Ethics Committee of Health Science Center, Xi’an Jiaotong University, and stated that no formal ethics approval was required for this type of study. All respondents were informed of the aim of the study and oral consent was obtained before participation.

## 3. Results

### 3.1. Availability

The mean availability of surveyed medicines in the public sector was 1.3% for originator brands (OBs) and 25.0% for lowest-priced generics (LPGs). Meanwhile, in the private sector, the availability increased to 34.7% for OBs and 35.4% for LPGs. The 37 medicines listed in the PEML had an availability of 30.1% for LPGs in the public sector, while availability was 38.5% for OBs and 41.3% for LPGs in the private sector.

Table 1 reports the availability of individual medicines in both sectors. Eight OBs found in the public sector had a very low availability, while 44 OBs were found in the private sector. No surveyed OBs had a high availability of more than 80% in either sector. Only seven LPGs in the public sector and 11 in the private sector had >50% availability. LPGs of amlodipine, losartan, and metformin had >80% availability in both the public and private sectors. Moreover, LPGs of atorvastatin, captopril and simvastatin were found to have high availability in the private sector (see Table A2 in Appendix A).

**Figure 1:**
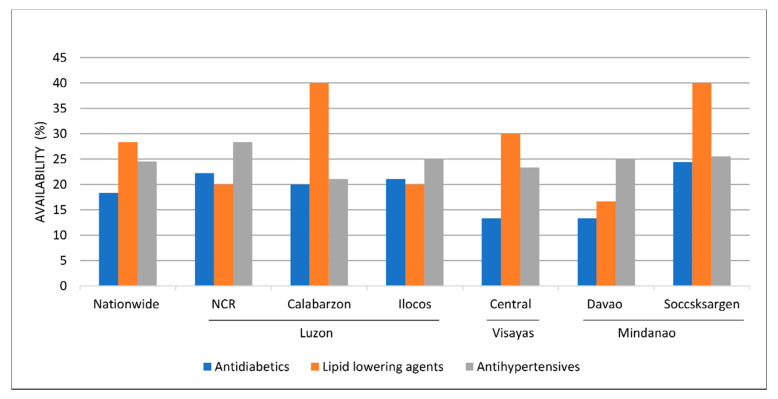
Availability of LPGs in the public sector per pharmacological category, nationwide and across regions. NCR, National Capital Region.

Figure 1 illustrates the availability of lowest-priced generics in public outlets across regions and pharmacological categories. Overall, antidiabetics had the lowest availability with 18.3%, especially in Central Visayas and Davao region. Lipid-lowering agents, on the other hand, had the highest availability with 28.3%, and the high availability of this category was evident in the Calabarzon and Soccsksargen regions. LPGs of antihypertensives had an availability of 24.5% in public outlets across the survey areas. However, it was still far below the ideal availability of 80% set by the WHO.

### 3.2. Prices

Table 1 presents the median unit price of individual medicines across sectors. MUP was not calculated when medicines were found in fewer than three outlets. Due to the availability of data, only 25 surveyed medicines were included in the comparative analysis of LPGs between public and private sectors, presented in Table 2. Beta-blockers had the highest price difference between OBs and LPGs in private outlets, with 6.44 for atenolol and 6.38 for propranolol, while valsartan has the smallest price difference with an OB/LPG of 1.31. Overall, the LPGs of amlodipine, furosemide, all human insulins (isophane, mixed, regular), losartan, losartan plus hydrochlorothiazide, metformin and simvastatin were higher priced in the private sector than in the public sector based on the *p*-value (*p* < 0.05). Meanwhile, the prices of other LPGs were not significantly different across both sectors (*p* > 0.05).

### 3.3. Affordability

Affordability of 20 standard treatments for diabetes, hypercholesterolemia and hypertension is presented in Table 3. All OB treatments’ costs were more than 1 day’s wage, except for gliclazide. Purchasing the lowest-priced generic medicines instead of OBs would be more affordable for the patients, especially from the public health sector. However, some LPGs were found to be more affordable in the private sector, such as methyldopa, nifedipine, propranolol, spironolactone and telmisartan. Among all the surveyed medicines, the OB of insulin glargine is the most unaffordable. It would need 7.88 days’ wages to pay for the monthly treatment. Moreover, fenofibrate and clonidine are the least affordable lipid-lowering agent and antihypertensive, respectively. 

**Figure 2:**
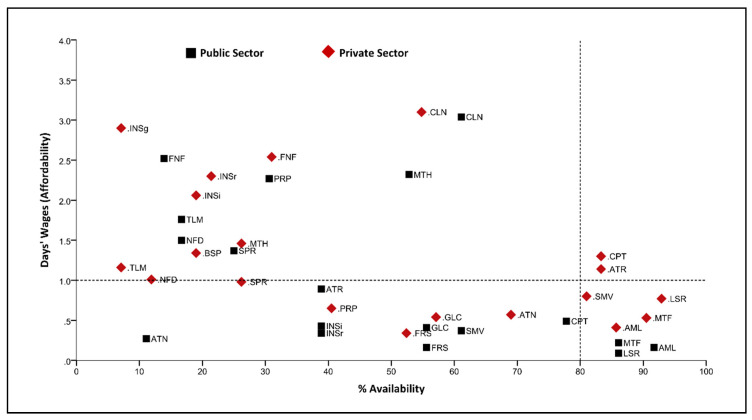
Comprehensive analysis of LPGs’ availability and affordability in public and private sector. AML, amlodipine; ATN, atenolol; ATR, atorvastatin; BSP, bisoprolol; CLN, clonidine; CPT, captopril; FNF, fenofibrate; FRS, furosemide; GLC, gliclazide; INSg, insulin glargine; INSi, insulin human isophane; INSr, insulin human regular; LSR, losartan; MTH, methyldopa; MTF, metformin; NFD, nifedipine; PRP, propranolol; SMV, simvastatin; SPR, spironolactone; TLM, telmisartan.

Figure 2 illustrates a comprehensive analysis of LPGs’ availability and affordability. In both sectors, LPGs of amlodipine, losartan and metformin, as well as simvastatin in the private sector, achieved the desired target of more than 80% availability and less than a day’s wage for affordability.

## 4. Discussion

Lack of access to medicines is an inequality that leads to pain, suffering and death from preventable diseases. To our knowledge, this is the first study in the Philippines to use a modified WHO/HAI methodology to describe the availability, price and affordability of VAT-exempt medicines. The results of the study and discussion is summarized and highlighted as follows, only four LPGs out of the 50 surveyed medicines met the WHO ideal target of 80% availability and less than one day’s wage in both public and private outlets. This shows the Filipino’s sub-optimal access to medicines despite the implementation of VAT exemption and lowered prices. The low availability and varied supply of most medicines in public outlets across different regions may be explained by the Philippines’ decentralized procurement system. The findings highlight the need for a shift of focus in policies from price reduction to improved availability of essential medicines for NCDs. 

This study has presented a lower availability of OBs than the generic alternatives in both public and private sectors; similar studies in developing countries have also demonstrated this aspect [24]. Surveys focusing on access to NCD medicines were recently published from low-middle income countries, the same income group with the Philippines. The findings show that the availability of NCD medicines in both Kenya and Zambia is significantly lower than the WHO ideal target of 80% [29,30]. Similarly, availability is poor in the public sector, and generally highest in the private for-profit sector.

In the Philippines, the Generics Law was already enacted in 1988 [8] but was strengthened through the provisions of the Universally Accessible and Quality Medicines Act in 2009 [9]. Through the years, the use of generic medicines was continually promoted and effectively implemented. Thus, the results in the study were coherent since the Generic Law mandates all public health institutions and public procurement to use generic alternatives in drug labeling, prescribing, dispensing and advertising.

As seen in Figure 1, LPGs’ availability in the public sector varies in different regions as medicine management in the Philippines happens through decentralization. The DOH is responsible for the procurement and management of medicines for its programs at the national level. Whereas, the local governments are in charge of purchasing and providing medicines not covered by the DOH, using their local budgets [31]. Reliable supply systems in public health entities are considered as a framework for improving access to essential medicines for NCDs [1]. With the low availability of antidiabetics, lipid-lowering agents and antihypertensives in the public sector, the patients are expected to purchase their medicines from private pharmacies, hence, increasing the patient’s out-of-pocket spending on drugs. The low availability of most “new medicines” in the private sector may be explained by low demand, as pharmacies may not stock these medicines. Moreover, the availability of insulin products was worth noting as both sectors had a low availability of <50%. The LPGs of all surveyed insulin in the private sector had a <30% availability, which is considered to be very low.

In the current study, the median price ratio (MPR) was not calculated due to the outdated 2015 MSH standard price reference. Therefore, a comparison with the international price ratio (IPR) was not done. However, the median price unit was presented in individual medicines. Some public sectors provide free medicines, while some add mark-up prices for storage and handling. Six LPGs in the public sector were found to be more expensive compared to the private sector, such as irbesartan plus hydrochlorothiazide, methyldopa, nifedipine, spironolactone, telmisartan, and valsartan. However, after statistical analysis, results revealed that the prices of these medicines do not have a significant difference with the private sector based on the *p*-value (*p* > 0.05). 

The private sector showed generally higher prices compared to the public sector, and OBs cost more than the LPGs. Similar findings were also seen in studies conducted in other developing countries [32,33,34]. Ball and Tisocki investigated high drug prices in the Philippines by examining the medicine price components [35]. They found that the significant contributor to the medicines’ high prices is the selling price set by the manufacturer. They also noted that a 12% VAT adds significantly to the cost of medicines.

The Philippine government has placed five drugs (amlodipine, atorvastatin, azithromycin, cytarabine, and doxorubicin) under MDRP and influenced pharmaceutical companies to reduce the prices of 16 OBs by half under the GMAP policy [36]. With the implementation of a 12% VAT exemption in antidiabetics, lipid-lowering agents and antihypertensives, we compared the prices of nine OBs under the current MDRP/GMAP and VAT-free prices in the private sector (see Table A3 in Appendix A). The data revealed a slight decrease in unit prices after the implementation, excluding irbesartan plus hydrochlorothiazide. 

A discussion paper by Reyes et al. presented a comparison of medicine prices included in MDRP/GMAP and prices retrieved from 2010 MIMS Philippines (Monthly Index of Medical Specialties) [37]. The findings revealed that drug prices correspond to the maximum prices imposed at the MDRP policy, and prices were decreased under the voluntary price reduction scheme of GMAP. Sarol reported the same findings when evaluating the effect of GMAP, using data obtained from independent surveys of IMS Health Philippines in 2009 and 2011 [38]. Therefore, the MDRP/GMAP policy has been effective as far as decreasing the prices of the selected medicines is concerned [37]. Yet, even with the new policy exempting the 12% VAT, there was only a minor decrease in the prices of the medicines for diabetes, hypercholesterolemia, and hypertension.

With regard to affordability, medicines are considered to be reasonably priced if the standard treatment costs one or less day’s wages of the lowest-paid unskilled government worker [19]. Using this criterion, all OBs available in the public sector were unaffordable, while 11 out of 18 LPGs shown in Table 3 were found to be affordable. In the private sector, all OBs were unaffordable except for gliclazide, and 9 out of 20 LPGs in the private sector were affordable.

The data can be compared with the latest studies conducted from other middle-income countries in Asia, i.e., Malaysia, China and Pakistan. Similar affordability of antihypertensive medicines (atenolol, captopril and furosemide) was seen in Malaysia’s private health sector, but the OB of propranolol was significantly higher in the Philippines requiring 4.2 days, while 0.8 days were required in Malaysia [39]. Insulin isophane and insulin regular were less affordable in the Philippines (3.9 days), as compared to China (1.1 days) and Pakistan (1.4 days) [33,40].

Moreover, we compared the affordability of four medicines (captopril, glibenclamide, metformin, and simvastatin) in the Philippines from 2009 and 2019; both studies used the WHO/HAI methodology (see Table A4 in Appendix A) [7]. It was noted that the days’ wages needed to purchase three LPGs medicines in 2009 were significantly reduced by more than a half in 2019. The data suggest that the OBs of metformin and simvastatin remain unaffordable in the private sector, requiring more than a day’s wage to purchase a monthly treatment of the disease. The OB of captopril has the highest number of days’ wages needed with 7.5 in 2009. However, data were not available in 2019 for comparison. Though the LPGs of these medicines became more affordable in the public sector, it has still poor availability, which hinders the patients from accessing them, especially glibenclamide. Low availability in the public sector means that patients have to purchase in the private sector, resorting to out-of-pocket spending. Medicines from the private sector are less affordable because of high prices and profit marginalization [32]. In this regard, policies monitoring the availability of essential medicines in the public sector should be considered and implemented.

The present study has several limitations. Data on medicine availability was collected at a specific time point and for a particular pharmacological category only. Thus, the data may not reflect the average medicine availability over time and may not be generalizable to medicines indicated for other diseases. Moreover, due to insufficient documentation, medicines included in the survey were not categorized based on the expected availability at a certain level of care in the public health sector. It may affect the average estimation of medicine availability. This study shows, however, the availability of surveyed medicines that the patients will encounter in all levels of the public health sector. 

The MPR was not calculated, and therefore, the data collected were not comparable with the international reference price. Results on affordability may also lead to over-estimation since the calculation used was based on the lowest-paid government workers’ wages. A significant proportion of the population earn less than the lowest-paid government worker. Lastly, the calculation of affordability utilizes the standard dose of individual medicines. Affordability may vary if patients are taking more than one drug. Regardless of the above limitations, this study is the first to evaluate the availability and affordability of VAT-exempt medicines in the Philippines and provides insight regarding the Filipino’s access to medicines.

## 5. Conclusions

Access to NCD medicines in both sectors was affected by low availability, which is lower than the 80% availability target set by WHO. The affordability of medicines was influenced by the high prices of originator brands even with VAT exemption. However, most LPGs were affordable in both sectors. Our findings show the Filipino’s sub-optimal access to medicines even with the implementation of VAT exemption and lowered prices. A review of policies and regulations should be initiated for better access to medicines in the Philippines.

## Figures and Tables

**Table 1:** Availability and median unit price of individual medicines in both the public and private sector.

Medicine Name	PEML	WHO/HAI LIST	Availability (%)				Median Unit Price (PHP)			
			OBs		LPGs		OBs		LPGs	
			PUB	PRV	PUB	PRV	PUB	PRV	PUB	PRV
acarbose	No	S	0.0	19.0	0.0	2.4	-	13.75	-	-
amlodipine	Yes	W	0.0	57.1	91.7	85.7	-	21.86	2.00	5.00
atenolol	Yes	W	0.0	14.3	11.1	69.0	-	45.10	3.29	7.00
atorvastatin	Yes	S	0.0	52.4	38.9	83.3	-	38.25	11.00	14.00
bisoprolol	Yes	G	0.0	45.2	2.8	19.0	-	26.79	-	16.50
candesartan	No	S	0.0	2.4	0.0	45.2	-	-	-	15.00
captopril	Yes	G	0.0	4.8	77.8	83.3	-	-	3.00	8.00
carvedilol	Yes	W	0.0	0.0	36.1	73.8	-	-	13.00	9.75
clonidine	Yes	S	11.1	59.5	61.1	54.8	27.13	32.25	18.67	19.05
dapagliflozin	No	S	2.8	38.1	0.0	0.0	-	52.00	-	-
empagliflozin	No	S	0.0	45.2	0.0	0.0	-	59.00	-	-
enalapril	Yes	S	0.0	14.3	5.6	54.8	-	43.75	-	14.00
enalapril + HCTZ	Yes	S	0.0	7.1	0.0	9.5	-	42.56	-	13.35
felodipine	Yes	S	0.0	35.7	16.7	33.3	-	38.50	12.50	13.30
fenofibrate	Yes	S	0.0	26.2	13.9	31.0	-	95.00	31.00	31.25
furosemide	Yes	W	0.0	64.3	55.6	52.4	-	21.00	2.00	4.13
glibenclamide	No	S	0.0	26.2	19.4	42.9	-	8.00	5.50	3.75
gliclazide	Yes	W	0.0	47.6	55.6	57.1	-	9.65	5.00	6.70
glimepiride	No	S	/	/	0.0	66.7	/	/	-	10.00
glipizide	No	S	0.0	19.0	2.8	14.3	-	27.38	-	12.68
hydralazine	Yes	W	/	/	0.0	0.0	/	/	-	-
hydrochloro-thiazide	Yes	W	/	/	0.0	21.4	/	/	-	6.45
indapamide	Yes	S	0.0	21.4	0.0	2.4	-	40.25	-	-
insulin glargine	No	S	2.8	23.8	2.8	7.1	-	2907.50	-	1068.66
insulin human isophane	Yes	W	0.0	31.0	38.9	19.0	-	1431.00	158.57	759.00
insulin human mixed *	Yes	S	0.0	28.6	30.6	19.0	-	1440.50	252.00	822.50
insulin human regular	Yes	W	2.8	33.3	38.9	21.4	-	1425.00	127.00	850.00
irbesartan	Yes	S	0.0	52.4	30.6	57.1	-	24.25	9.00	15.75
irbesartan + HCTZ	Yes	S	0.0	47.6	11.1	16.7	-	31.50	21.88	19.50
linagliptin	No	S	0.0	54.8	0.0	0.0	-	60.75	-	-
lisinopril	No	S	0.0	11.9	0.0	2.4	-	67.50	-	-
losartan	Yes	W	0.0	42.9	86.1	92.9	-	19.35	1.15	9.50
losartan + HCTZ	Yes	S	0.0	28.6	30.6	69.0	-	22.63	6.00	12.50
metformin	Yes	G	0.0	45.2	86.1	90.5	-	14.25	1.34	3.25
methyldopa	Yes	W	8.3	54.8	52.8	26.2	24.00	22.75	14.29	9.00
metoprolol	Yes	W	/	/	58.3	78.6	/	/	2.00	3.25
nifedipine	Yes	S	0.0	19.0	16.7	11.9	-	44.40	18.48	12.42
nimodipine	Yes	S	0.0	26.2	5.6	2.4	-	45.75	-	-
pioglitazone	No	S	0.0	7.1	0.0	40.5	-	67.00	-	12.70
pravastatin	No	S	/	/	0.0	23.8	/	/	-	25.78
propranolol	Yes	W	0.0	47.6	30.6	40.5	-	25.50	13.97	4.00
rosuvastatin	Yes	S	0.0	42.9	27.8	64.3	-	85.93	20.50	20.50
simvastatin	Yes	G	0.0	23.8	61.1	81.0	-	33.88	4.49	9.83
sitagliptin	No	S	0.0	40.5	0.0	0.0	-	60.00	-	-
spironolactone	Yes	W	5.6	52.4	25.0	26.2	-	18.50	16.83	12.00
telmisartan	Yes	S	13.9	73.8	16.7	7.1	24.50	25.00	21.62	14.25
telmisartan + HCTZ	Yes	S	2.8	59.5	0.0	4.8	-	24.85	-	-
valsartan	Yes	S	0.0	47.6	13.9	38.1	-	25.25	22.14	19.24
valsartan + HCTZ	Yes	S	0.0	33.3	0.0	14.3	-	26.80	-	23.48
verapamil	Yes	S	0.0	31.0	0.0	14.3	-	93.50	-	42.46

PEML, Philippine Essential Medicines List; S, Supplementary List; W, WHO Model List of Essential Medicines; G, WHO Global Core List; OBs, Originator Brands; LPGs, Lowest-priced Generics; PUB, public medicine outlet; PRV, private medicine outlet; PHP, Philippine Peso; HCTZ, hydrochlorothiazide; mixed *, 70% isophane + 30% regular; (/) This brand is off-market in the Philippines and not included in the analysis; (-) No data available.

**Table 2:** Comparison of LPGs’ median unit price (in PHP; Philippine peso) between public and private sectors.

Medicine Name	Public Sector	Private Sector			Comparison between Public and Private Sectors
	LPGs	OBs	LPGs	OBs/LPG	*p*-Value for LPGs *
amlodipine	2.00	21.86	5.00	4.36	0.000
atenolol	3.29	45.10	7.00	6.44	0.290
atorvastatin	11.00	38.25	14.00	2.73	0.417
clonidine	18.67	32.25	19.05	1.69	0.339
felodipine	12.50	38.50	13.30	2.89	0.741
fenofibrate	31.00	95.00	31.25	3.04	0.758
furosemide	2.00	21.00	4.13	5.08	0.006
glibenclamide	5.50	8.00	3.75	2.13	0.671
gliclazide	5.00	9.65	6.70	1.44	0.106
insulin human isophane	158.57	1431.00	759.00	1.89	0.000
insulin human mixed ^%^	252.00	1440.50	822.50	1.75	0.004
insulin human regular	127.00	1425.00	850.00	1.68	0.001
irbesartan	9.00	24.25	15.75	1.54	0.194
irbesartan + HCTZ	21.88	31.50	19.50	1.62	0.850
losartan	1.15	19.35	9.50	2.04	0.000
losartan + HCTZ	6.00	22.63	12.50	1.81	0.009
metformin	1.34	14.25	3.25	4.38	0.000
methyldopa	14.29	22.75	9.00	2.53	0.219
nifedipine	18.48	44.40	12.42	3.57	0.715
propranolol	13.97	25.50	4.00	6.38	0.221
rosuvastatin	20.50	85.93	20.50	4.19	0.600
simvastatin	4.49	33.88	9.83	3.45	0.000
spironolactone	16.83	18.50	12.00	1.54	0.380
telmisartan	21.62	25.00	14.25	1.75	0.236
valsartan	22.14	25.25	19.24	1.31	0.943

LPGs, Lowest-priced Generics; OBs, Originator Brands; HCTZ, hydrochlorothiazide; mixed ^%^, 70% isophane + 30% regular; *, *t* test or ANOVA for data with normal distribution and Mann–Whitney U test for data with non-normal distribution.

**Table 3:** Affordability of standard treatments purchased by the lowest-paid government worker.

Medicine Name	Strength	Dosage Form	No. of Units Per Day	Total No. of Units Per Month	Day’s Wages to Pay for Treatment			
					Public Sector		Private Sector	
					OBs	LPGs	OBs	LPGs
Antidiabetics								
gliclazide	80 mg	cap/tab	1	30	-	0.4	0.8	0.5
insulin glargine	100 IU/mL	10 mL vial	-	10 mL	-	-	7.9	2.9
insulin isophane	100 IU/mL	10 mL vial	-	10 mL	-	0.4	3.9	2.1
insulin regular	100 IU/mL	10 mL vial	-	10 mL	-	0.3	3.9	2.3
metformin	500 mg	cap/tab	2	60	-	0.2	2.3	0.5
Lipid-lowering Agents								
atorvastatin	20 mg	cap/tab	1	30	-	0.9	3.1	1.1
fenofibrate	160 mg	cap/tab	1	30	-	2.5	7.7	2.5
simvastatin	20 mg	cap/tab	1	30	-	0.4	2.8	0.8
Antihypertensives								
amlodipine	5 mg	cap/tab	1	30	-	0.2	1.8	0.4
atenolol	50 mg	cap/tab	1	30	-	0.3	3.7	0.6
bisoprolol	5 mg	cap/tab	1	30	-	-	2.2	1.3
captopril	25 mg	cap/tab	2	60	-	0.5	-	1.3
clonidine	75 mcg	cap/tab	2	60	4.4	3.0	5.2	3.1
furosemide	40 mg	cap/tab	1	30	-	0.2	1.7	0.3
losartan	50 mg	cap/tab	1	30	-	0.1	1.6	0.8
methyldopa	250 mg	cap/tab	2	60	3.9	2.3	3.7	1.5
nifedipine	30 mg	SR cap/tab	1	30	-	1.5	3.6	1.0
propranolol	40 mg	cap/tab	2	60	-	2.3	4.2	0.7
spironolactone	25 mg	cap/tab	1	30	-	1.4	1.5	0.9
telmisartan	40 mg	cap/tab	1	30	2.0	1.8	2.0	1.2

## Appendix A

**Table A1:** Information of survey areas and number of sample facilities.

Island Group	Region	Population (million)	GRDP Per Capita ($)	Surveyed Public Outlets	Surveyed Private Outlets
Luzon (*n* = 3)	National Capital Region	13.05 (12.2%)	9579.73	6	7
	Calabarzon Region	14.92 (14.0%)	3295.13	6	7
	Ilocos Region	5.33 (5.0%)	1966.23	6	7
Visayas (*n* = 1)	Central Visayas Region	7.81 (7.3%)	2831.52	6	7
Mindanao (*n* = 2)	Davao Region	5.25 (4.9%)	2976.67	6	7
	Soccsksargen Region	4.87 (4.6%)	1855.60	6	7
			**TOTAL**	**36**	**42**

Source: Philippine Statistics Authority 2018; %, percent share from the total population in the Philippines; GRDP, Gross Regional Domestic Product.

**Table A2:** Availability of medicines in the public and the private sectors.

Availability	Public Sector		Private Sector	
	Originator Brand	Lowest-Priced Generic	Originator Brand	Lowest-Priced Generic
**Medicines not found in any outlets (0%)**	all excluding the eight medicines listed below (*n* = 37)	acarbose, candesartan, dapagliflozin, empagliflozin, enalapril + HCTZ, glimepiride, hydralazine, hydrochlorothiazide, indapamide, linagliptin, lisinopril, pioglitazone, pravastatin, sitagliptin, telmisartan + HCTZ, valsartan + HCTZ, verapamil (*n* = 17)	carvedilol (*n* = 1)	dapagliflozin, empagliflozin, hydralazine, linagliptin, sitagliptin (*n* = 5)
**Medicines with very low availability (<30%)**	clonidine, dapagliflozin, insulin glargine, insulin human regular, methyldopa, spironolactone, telmisartan, telmisartan + HCTZ (*n* = 8)	atenolol, bisoprolol, enalapril, felodipine, fenofibrate, glibenclamide, glipizide, insulin glargine, irbesartan + HCTZ, nifedipine, nimodipine, rosuvastatin, spironolactone, telmisartan, valsartan (*n* = 15)	acarbose, atenolol, candesartan, captopril, enalapril, enalapril + HCTZ, fenofibrate, glibenclamide, glipizide, indapamide, insulin glargine, insulin human mixed, lisinopril, losartan + HCTZ, nifedipine, nimodipine, pioglitazone, simvastatin (*n* = 18)	acarbose, bisoprolol, enalapril + HCTZ, glipizide, hydrochlorothiazide, indapamide, insulin glargine, insulin human isophane, insulin human mixed, insulin human regular, irbesartan + HCTZ, lisinopril, methyldopa, nifedipine, nimodipine, pravastatin, spironolactone, telmisartan, telmisartan + HCTZ, valsartan + HCTZ, verapamil (*n* = 21)
**Medicines with low availability (30–49%)**	None	atorvastatin, carvedilol, insulin human isophane, insulin human mixed, insulin human regular, irbesartan, losartan + HCTZ, propranolol (*n* = 8)	bisoprolol, dapagliflozin, empagliflozin, felodipine, gliclazide, insulin human isophane, insulin human regular, irbesartan + HCTZ, losartan, metformin, propranolol, rosuvastatin, sitagliptin, valsartan, valsartan + HCTZ, verapamil (*n* = 16)	candesartan, felodipine, fenofibrate, glibenclamide, pioglitazone, propranolol, valsartan (*n* = 7)
**Medicines with fairly high availability (50–80%)**	None	captopril, clonidine, furosemide, gliclazide, methyldopa, metoprolol, simvastatin (*n* = 7)	amlodipine, atorvastatin, clonidine, furosemide, irbesartan, linagliptin, methyldopa, spironolactone, telmisartan, telmisartan + HCTZ (*n* = 12)	atenolol, carvedilol, clonidine, enalapril, furosemide, gliclazide, glimepiride, irbesartan, losartan + HCTZ, metoprolol, rosuvastatin (*n* = 11)
**Medicines with high availability (>80%)**	None	amlodipine, losartan, metformin (*n* = 3)	None	amlodipine, atorvastatin, captopril, losartan, metformin, simvastatin (*n* = 6)

**Table A3:** Comparison of prices in MDRP/GMAP to the current MUP.

Medicine Name	Brand Name	Dosage Strength	Price in MDRP/ GMAP (PHP)	Median Unit Price 2019 (PHP)
amlodipine *	Norvasc	5 mg	22.85	21.86
atorvastatin *	Lipitor	20 mg	39.13	38.25
gliclazide	Diamicron	80 mg	9.75	9.65
irbesartan	Aprovel	150 mg	24.38	24.25
irbesartan + HCTZ	Coaprovel	150 mg + 12.5 mg	25.13	31.50
losartan	Cozaar	50 mg	21.50	19.35
losartan + HCTZ	Hyzaar	50 mg + 12.5 mg	23.75	22.63
telmisartan	Micardis	40 mg	25.75	25.00
telmisartan + HCTZ	Micardis Plus	40 mg + 12.5 mg	25.00	24.85

* Covered by Maximum Drug Retail Price (MDRP) scheme. All others are under the Government-Mediated Access Price (GMAP) scheme.

**Table A4:** Comparison of affordability of medicines in the Philippines.

Medicines	Public		Private			
	LPGs		LPGs		OBs	
	2009	2019	2009	2019	2009	2019
**Antihypertensives**						
captopril	1.1	0.5	1.9	0.6	7.5	-
**Antidiabetics**						
glibenclamide	0.4	0.5	1.1	1.3	2.4	0.7
metformin	1.0	0.2	1.4	0.5	3.6	2.3
**Lipid-lowering Agent**						
simvastatin	1.3	0.4	2.1	0.8	3.4	2.8
